# Diagnostic dilemma: late presentation of amelanotic BRAF-negative metastatic malignant melanoma resembling clear cell sarcoma – a case report

**DOI:** 10.1186/1746-1596-8-192

**Published:** 2013-11-25

**Authors:** Barrett Parker Wagner, Narendranath Epperla, Rafael Medina-Flores

**Affiliations:** 1 University of Wisconsin School of Medicine and Public Health, 750 Highland Ave, Madison, WI, USA; 2 Department of Internal Medicine and Clinical Research, Marshfield Clinic, Marshfield Clinic Research Foundation, 1000 N Oak Ave, Marshfield, WI, USA; 3 Department of Pathology, Marshfield Clinic, 1000 N Oak Ave, Marshfield, WI, USA

**Keywords:** Malignant melanoma, Clear cell sarcoma (of tendons and aponeuroses)

## Abstract

Clear cell sarcoma is a rare cancer primarily of tendons, fascia, and aponeuroses that can be difficult to discern from primary cutaneous malignant melanoma. The two cancers share several histological markers, with most cases of both cancers staining positively for S-100, HMB-45, and melanin. Primary therapy of both cancers involves wide local excision, but while systemic therapy has proven benefit for malignant melanoma, it has not been established for clear cell sarcoma.

We report the case of a 58 year old woman with a large, ulcerated, fungating mass on her left lower leg. Frozen section of the mass showed a malignant epithelioid and spindle cell tumor confined to the subcutaneous tissue. A provisional diagnosis of soft-tissue sarcoma was made. Through in-depth study of initial biopsy with immunohistochemistry for S-100, HMB-45, MART-1, and MITF, along with karyotyping and FISH analysis for EWS gene rearrangement, the diagnosis of amelanotic malignant melanoma was confirmed. The patient then underwent systemic treatment with ipilimumab upon recurrence with good response.

**Virtual slides:**

The virtual slide(s) for this article can be found here: http://www.diagnosticpathology.diagnomx.eu/vs/1989338475107348.

## Introduction

Primary cutaneous malignant melanoma (MM) is an aggressive cancer of melanocytes that causes 75% of skin cancer deaths [1]. The presentation can vary greatly, with many different types. These include the four common subtypes of lentigo maligna, superficial spreading melanoma, acral-lentiginous melanoma, and nodular melanoma, as well as the rarer desmoplastic melanoma and mucosal melanoma [2]. The prognosis for a patient with MM depends greatly on the tumor stage.

Primary cutaneous malignant melanoma can be very similar to, but is distinct from, clear cell sarcoma of tendons and aponeuroses (CCSTA) [3]. CCSTA originates chiefly from tendons, aponeuroses, and fascia of the extremities; most often beginning in the feet or knees of young adults [2,4]. The cells of origin are suspected to be of neural crest origin due to evidence of melanocyte differentiation in many tumors [5]. Further hindering discrimination from MM, CSSTA cells sometimes produce melanin, and have been known to extend into the subcutis and dermis [4]. It is important to distinguish these two conditions not only due to the difference in prognosis but also due to difference in treatment. Though the primary treatment for both these conditions is wide-margin resection, systemic therapy is beneficial for those with advanced or metastatic MM, in contrast to CCSTA, where there is no established benefit to chemotherapy.

We present the case of a 58 year old female who posed a diagnostic challenge, as her lesion was initially considered to be a soft tissue sarcoma based on gross morphology and imaging findings of the mass. However, after a detailed work-up she was diagnosed with amelanotic malignant melanoma and responded to systemic therapy.

## Case presentation

### Patient course

A 58 year old woman presented to the emergency department with a bleeding mass on the medial aspect of her lower left leg. Three years prior, she sustained a burn to that area of her leg resulting in a scar. One year ago, she sustained minor trauma to that region leading to a small painless bump that slowly progressed in size. She noticed bleeding from the mass, which prompted her to seek medical attention. Upon presentation to the emergency department, the bleeding had stopped.

The patient denied constitutional symptoms of fever, chills and night sweats or recent weight loss. She had no numbness, tingling or localized weakness. There was no history of cancer in the family. The patient was married and worked as a sales clerk. She was a non-smoker and did not drink alcohol.

Examination showed a multiloculated and fungating soft tissue mass measuring 15 cm × 18 cm × 5 cm, with interspersed areas grossly consistent with necrosis. Neurologic function was normal distal to the mass with intact ability to flex and extend both the ankle and toes painlessly. A 6–8 cm non-fungating, palpable mass was also noted in the left thigh near the groin. Both masses were non-tender. In addition, right supraclavicular and bilateral axillary lymphadenopathy was appreciated.

**Figure 1:**
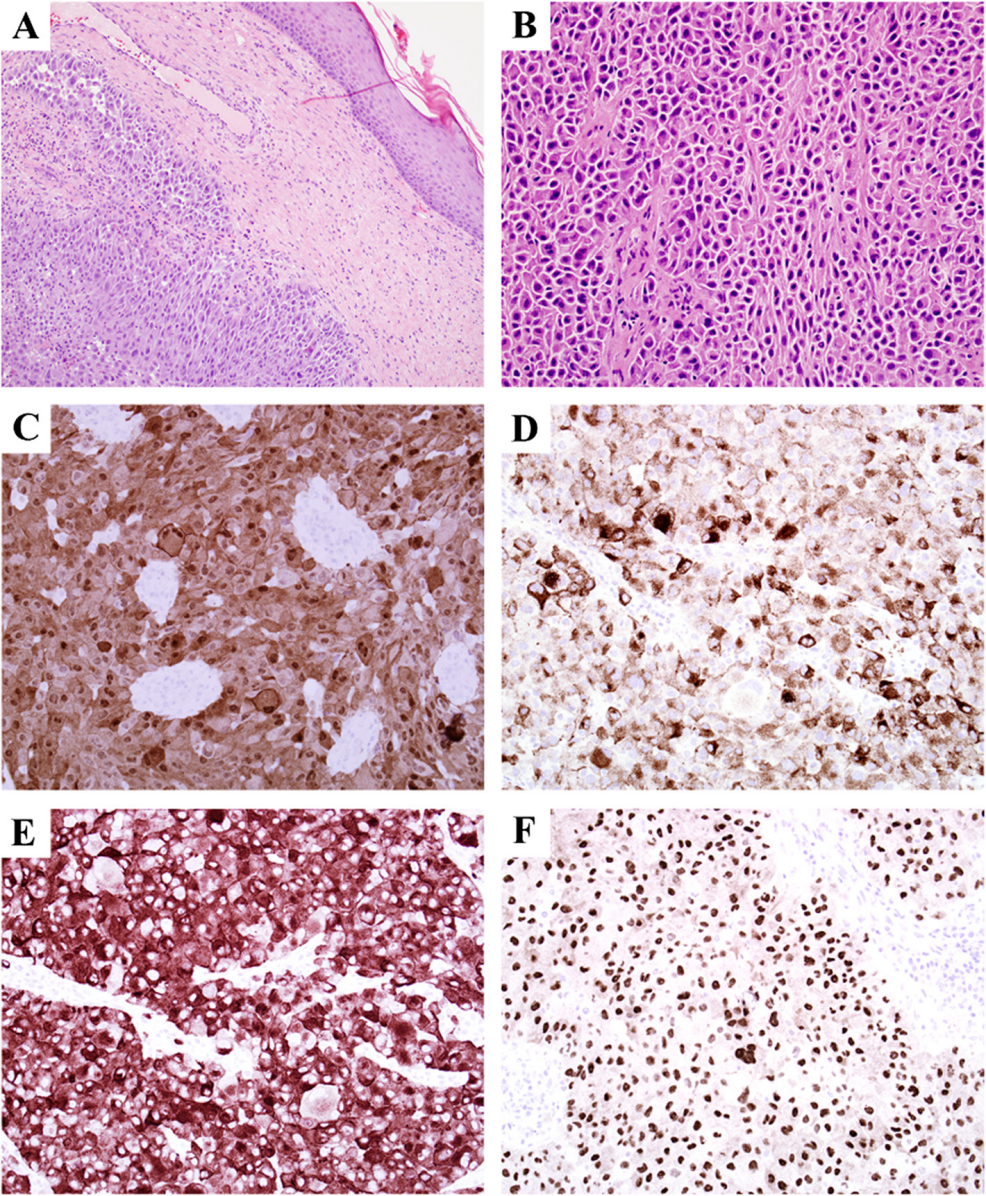
**Immunostains of tissue biopsy. (A)**: 10× H&E; the dermis was infiltrated by sheets and nests of tumor cells with eosinophilic cytoplasm and large nuclei with prominent nucleoli, the epidermis was uninvolved. **(B)**: 20× H&E; higher power of tumor within subcutis shows a nested tumor architecture, neoplastic cells are somewhat discohesive and have bright eosinophilic cytoplasm. **(C)**: 20× S-100; positive nuclear and cytoplasmic reactivity in tumor cells for S-100. **(D)**: 20× HMB-45; positive cytoplasmic reactivity. **(E)**: 20× Melan-A; positive cytoplasmic reactivity. **(F)**: 20× MITF; positive nuclear reactivity.

The mass was suspected to be a soft tissue sarcoma. Staging abdominal, pelvic, and chest CT was performed and the patient underwent surgical amputation of the left leg below the knee, as well as excision of masses in the following locations: left groin (16 cm), right supraclavicular (3 cm), left anterior shoulder (3 cm), right wrist, left mid-back, right posterior axilla, and right lateral breast. The patient tolerated the lengthy procedure well. Biopsy of the mass showed sheets and nests of epithelioid and spindle tumor cells within the superficial and deep dermis and subcutis, with the epidermis uninvolved. On permanent section, tumor cells showed no evidence of melanin pigment on Fontana Masson stain (not shown), but S-100 protein, HMB-45, MART-1, and MITF were all positive in the tumor (Figure 1), which resulted in a histopathological diagnosis of malignant melanoma. At the request of the patient’s oncologist, additional fluorescence-in-situ hybridization (FISH) for Ewing Sarcoma Breakpoint Region (EWSR) was performed and was negative for a rearrangement. In addition, molecular testing for BRAF exon 15, was negative for the V600E mutation.

Although the primary site could not be discerned with certainty, assuming that the leg mass is the primary tumor yielded a stage of T4bN3M1a. Following discharge, the patient had monthly follow-ups with orthopedics, plastic surgery, and prosthetics. Six months after the original diagnosis, follow-up PET scan showed small lesions in the liver, spleen, retro-peritoneal lymph nodes and left external iliac nodes with increased activity. The patient began treatment with ipilimumab at that time, and was responsive to therapy.

## Discussion

This patient presented with an unusually large tumor on the lower leg, which she had allowed to grow for approximately 1 year. Due to the physical appearance and large presenting size, the diagnosis of sarcoma was made. The tumor location near the knee with a biopsy showing an uninvolved epidermis, positive staining with S-100 and HMB-45, and lack of melanin suggested a possibility of CCSTA. However, with more in-depth testing, the proper diagnosis of amelanotic malignant melanoma was confirmed.

Melanoma is the most aggressive of the cutaneous malignancies, causing more than 9,000 deaths in the past year in the United States. It is responsible for 80% of deaths from skin cancer despite accounting for only 4% of all dermatological cancers [6]. Abnormal growth of melanocytes in MM is due to activation of the mitogen-activated protein kinase (MAPK) via various mechanisms such as N-RAS or BRAF mutations [7]. Diagnosis is typically made histologically, but recent studies have shown promise for application of FISH for differentiating between nevi and several melanoma types [8,9]. The prognosis and tumor behavior varies according to the stage of the melanoma. In stage I and II (localized) melanomas, the best survival predictors are tumor thickness, mitotic rate and ulceration [10]. The prognosis of stage III melanomas is extremely variable depending on tumor burden, number of involved nodes, and ulceration. 5 year survival rates of such patients can vary from 13 to 69% [11]. Stage IV melanoma carries a grim prognosis, but those with metastasis to non-visceral sites and/or the lungs have better 1 year survival rates than those with metastasis to other visceral organs [11]. Furthermore, recent biomarker research has shown that high MCM3 expression is a sign of poor prognosis that correlates with reduced RBM3 expression [12].

Clear cell sarcoma is a rare but distinct clinic-pathologic entity, which predominantly affects tendons and aponeuroses of adolescents and young adults. It was first described in 21 cases in 1965 and given the name clear cell sarcoma of tendons and aponeuroses [13]. It later also became known as malignant melanoma of the soft parts, although this name has fallen out of favor as it has become more clear that the cells of origin are not melanocytes [3]. The tumor tends to remain localized, with tumor size being the primary determinant of prognosis.

For CSSTA, surgical resection with wide local margins is the primary treatment, resulting in 5 and 10 year survival rates of 59% and 41% respectively [14]. If margins are close, then local radiotherapy is indicated. There is currently not established benefit to chemotherapy for CSSTA, but in aggressive cases adjuvant therapy is often used [3]. Primary treatment of MM is wide-margin surgical resection. Sentinel lymph node biopsy is recommended for patients with lesions larger than 1 mm, as well as those with lesions that are ulcerated or have high mitotic rate [15,16]. Definitive diagnosis from the nodes is made with fixation and staining with hemotoxylin and eosin, along with immunohistochemical stains such as S-100, MART-1, and HMB-45 [17]. In contrast to CSSTA, systemic therapy has proven benefit for those with advanced or metastatic MM. Options include dacarbazine, temozolomide, IL-2, paclitaxel, cisplatin, carboplatin, IFN-α2b, and ipilimumab [15].

MM and CCSTA are very similar histologically, which led Chung and Enzinger to propose “malignant melanoma of soft parts” as a preferred name for clear cell sarcoma in 1983 [4]. The two are often difficult to differentiate because 70-90% of clear cell sarcomas express melanin due to fusion of the EWS and ATF1 genes from chromosomes 22q12 and 12q13 respectively. Further complicating differentiation between the two cancers is the fact that histological samples of both CCSTA and MM will stain positively for S-100 and HMB-45 [2]. Proper identification can be attained through use of MART-1 and MITF melanoma stains, and via FISH to detect EWS gene rearrangement [18]. Also, diagnosis of malignant melanoma can be made if a BRAF mutation is found [14]. This case was particularly difficult to diagnose due to the lack of V600E BRAF mutation and negative EWS rearrangement, either of which could have greatly clarified the diagnosis.

## Conclusions

While sometimes difficult to distinguish, clear cell sarcoma can be differentiated from malignant melanoma by examining a combination of primary tumor location, dissemination, growth speed, staining with MART-1 and MITF, and FISH detection of EWS gene rearrangement. This case presented a diagnostic challenge due to a number of factors: the patient fit the epidemiology of CSSTA (younger adult, tumor of the lower leg), the massive and hemorrhaging nature of the leg tumor masked whether the tumor origin cells came from the skin or soft tissue, and histologic markers of CSSTA and MM are very similar.

In the end, definitive diagnosis was made due to the fast-growing and widely disseminated nature of the cancer, positive staining with MART-1 and MITF, and FISH analysis revealing normal EWS gene. Although the original resection was performed before the definitive diagnosis was made, the thorough analysis eventually led to the proper diagnosis of MM, which allowed for treatment with ipilimumab upon recurrence with good response.

## Consent

Written informed consent was obtained from the patient for publication of this Case Report and any accompanying images. A copy of the written consent is available for review by the Editor-in-Chief of this journal.

## Abbreviations

MM: Malignant melanoma; CSSTA: Clear cell sarcoma (of tendons and aponeuroses); HMB-45: Human melanocyte black 45; MART-1: Melanocyte antigen recognized by T-cells 1; MITF: Microphthalmia-associated transcription factor; FISH: Fluorescence in-situ hybridization; EWSR: Ewing sarcoma breakpoint region.

## Competing interests

The authors declare that they have no competing interests.

## Authors’ contributions

BW performed chart review, literature review, and primary composure of written case report. NE performed chart review, literature review, correspondence with treatment team, and made substantial contribution to composure of the written case report. RMF performed histologic analysis, provided histologic images, and contributed to editing of the written case report. All authors read and approved the final manuscript.

## Authors’ information

BW is an MD candidate in the class of 2014 at the University of Wisconsin School of Medicine and Public Health in Madison, WI. NE is a hospitalist and clinical researcher at Marshfield Clinic/St. Joseph’s Hospital in Marshfield, WI. RMF is pathologist with expertise in anatomic pathology and neuropathology at Marshfield Clinic in Marshfield, WI.
